# The performance of the European League Against Rheumatism/American College of Rheumatology idiopathic inflammatory myopathies classification criteria in an expert-defined 10 year incident cohort

**DOI:** 10.1093/rheumatology/key343

**Published:** 2018-11-28

**Authors:** Matthew J S Parker, Alexander Oldroyd, Mark E Roberts, James B Lilleker, Zoe E Betteridge, Neil J McHugh, Ariane L Herrick, Robert G Cooper, Hector Chinoy

**Affiliations:** 1 Rheumatology Department, Salford Royal NHS Foundation Trust, Manchester Academic Health Science Centre, Salford, UK; 2 Centre for Musculoskeletal Research, Manchester Academic Health Science Centre, University of Manchester, Manchester, UK; 3 Rheumatology Department, Institute of Rheumatology and Orthopaedics, Royal Prince Alfred Hospital, Sydney, Australia; 4 Manchester Academic Health Science Centre, Greater Manchester Neurosciences Centre, Salford Royal NHS Foundation Trust, Salford, UK; 5 Department of Pharmacy and Pharmacology, University of Bath, Bath, UK; 6 Royal National Hospital for Rheumatic Disease, Bath, UK; 7 MRC-ARUK Centre for Integrated Research into Musculoskeletal Ageing, University of Liverpool, Liverpool, UK; 8 Centre for Integrated Genomic Medical Research, Division of Population Health, Health Services Research and Primary Care, Faculty of Biology, Medicine and Health, Manchester Academic Health Science Centre, University of Manchester, Manchester, UK; 9 NIHR Manchester Musculoskeletal Biomedical Research Centre, Manchester University Hospitals NHS Foundation Trust, Manchester Academic Health Science Centre, Manchester, UK

**Keywords:** idiopathic inflammatory myopathies, myositis, dermatomyositis, polymyositis, inclusion body myositis, classification

## Abstract

**Objectives:**

To assess the performance of the EULAR/ACR idiopathic inflammatory myopathies (IIMs) classification criteria in a cohort of incident IIM cases and examine how criteria-assigned IIM subtype correlates with expert opinion.

**Methods:**

Adults with newly diagnosed IIM attending Salford Royal NHS Foundation Trust were identified over a 10 year period. A retrospective review of all putative cases was performed and those fulfilling a consensus expert opinion diagnosis of IIM were included. Clinical, serological and histological data were collected and each case was assigned a single IIM subtype. The EULAR/ACR classification criteria were then applied and sensitivity, specificity and positive and negative predictive values were calculated, presented with 95% CIs.

**Results:**

A total of 1637 cases were screened, with 255 consensus expert opinion IIM cases ultimately identified. Applying the EULAR/ACR classification criteria, the sensitivity to diagnose an IIM was 99.6% (95% CI 97.2, 100) and 80.9% (95% CI 76.0, 85.8) for the criteria cut-points of probable and definite diagnoses, respectively. In 94/255 cases the IIM subtype differed between consensus expert opinion and classification criteria, most strikingly in the group subtyped as PM by the EULAR/ACR criteria, where there was discrepancy in the majority (i.e. in 87/161).

**Conclusion:**

The EULAR/ACR criteria performed with high sensitivity in identifying IIM in this external cohort of incident IIM. However, substantial disagreements arose between consensus expert opinion and the criteria regarding IIM subtype assignments, resulting in a large proportion of criteria-assigned cases of PM having heterogeneous features. These results may have important implications for future use of these criteria in subsequent research.

Rheumatology key messages

- EULAR/ACR idiopathic inflammatory myopathies (IIMs) classification criteria perform with high sensitivity in a newly diagnosed IIM external cohort.
- However, in 94/255 cases, the decision on IIM subtype differed between experts and the criteria.
- A non-diagnostic muscle biopsy, if included in the calculations, can reduce the sensitivity of the criteria.

## Introduction

The first widely accepted classification criteria for idiopathic inflammatory myopathies (IIMs) were published in 1975, but challenges remain when applying classification or diagnostic criteria to IIMs [1]. This is at least in part because the IIMs are inherently heterogeneous in their organ-specific manifestations as well as their severity, disease trajectory and response to treatment, even within well-defined disease subtypes [2]. Recent research has progressed our understanding of disease pathogenesis, genetic associations, molecular pathways, clinical subtypes, histological features and autoantibody associations [3]. Translating this contemporary progress into clinical practice has occurred more readily than has been reflected in the development of updated diagnostic and classification criteria needed to identify and stratify patients for subsequent research studies. However, given the tight interdependent relationship between clinical progress and research, especially in conditions as rare as IIM, it is important to ensure classification criteria remain updated and as clinically relevant as possible. These priorities have been well summarized by Lundberg *et al.* [4].

In response to this challenge, a 10 year international collaboration has led to the publication of new EULAR/ACR IIM classification criteria [5, 6]. After evaluating the role of multiple putative variables in the expert diagnosis of IIM, 16 variables were selected and given an individual weight score. The gross sum of these can be used directly or converted into a probability (as a percentage) of an IIM diagnosis. A second function of the EULAR/ACR IIM classification criteria is in the identification of distinct subtypes, of particular importance as they are known to have differing clinical phenotypes, natural histories and treatment responses. The criteria, when used with the accompanying classification tree, categorize cases into PM, DM, IBM or amyopathic DM (ADM).

Despite the achievement and progress that the EULAR/ACR criteria represent, a number of issues remain, many of them acknowledged by the authors [6]. There was a high frequency of missing data in both the derivation and validation samples. All cases used had established IIM with disease for at least 6 months (mean 3.0 years after diagnosis), but criteria were not evaluated at disease diagnosis, which may be an important time point for certain research purposes. Critically, when the data acquisition underpinning the development of the EULAR/ACR criteria started, there was only limited and locally varied availability of myositis-specific and associated autoantibody testing. As a result, the EULAR/ACR criteria have only included the anti-Jo-1 antibody. Additional IIM subtypes are now well established and include anti-synthetase syndrome (ASS; characterized by the presence of one of eight described anti-tRNA synthetase antibodies), immune-mediated necrotizing myopathy (IMNM; characterized by specific biopsy and/or autoantibody status) and overlap myositis (OM; where a relevant associated connective tissue disorder is also present, such as MCTD, SSc or RA [7–10]. Additionally, the EULAR/ACR criteria do not incorporate data relating to the presence of interstitial lung disease, an organ-specific manifestation accounting for a major component of morbidity and mortality and which can be the predominant or even sole clinical manifestation in IIM [11].

In an effort to evaluate the effect of these issues on the utility of the EULAR/ACR criteria, we examined the performance of the EULAR/ACR criteria in an external real-world cohort using otherwise unselected consecutive incident IIM cases collected over a 10 year period at our tertiary neuromuscular service. The secondary aim was to deeply phenotype cases and examine how classification criteria–assigned IIM subtype correlated with expert opinion.

## Materials and methods

This study forms part of a national quality improvement project aimed at identifying IIM cases for specialized disease commissioning, as accurate data will help to inform future service planning. Given this context, approval for the conduct of the project was granted without a recommendation to seek more formal ethics authorization, in keeping with local policy.

### Case identification

Salford Royal NHS Foundation Trust (SRFT) provides tertiary neuromuscular services, including for adults with IIM, for the Greater Manchester region, UK. Its long-standing status as a specialist neuromuscular service with advanced information technology systems makes it well suited to this study.

Adults (⩾18 years at disease onset) first diagnosed with IIM when attending the SRFT between 1 January 2007 and 31 December 2016 were eligible. Three stages of overlapping case ascertainment were employed. First, a broad International Classification of Diseases, 10th Revision coding search was performed for all in-patient hospital episodes relating to IIM at the SRFT during the study period. The codes selected were consistent with a recent epidemiological study and are included in the supplemental material, available at *Rheumatology* online [12]. Second, all new patients referred to the SRFT adult neuromuscular outpatient clinics were manually identified. Third, all SRFT patients with a positive myositis-specific antibody (MSA) result from serological studies requested by SRFT clinicians within the study period were identified. Excepting anti-Jo-1 antibodies, prior to 2014 all MSA requests were sent to and processed at the Bath Institute for Rheumatic Diseases serology service (UK), using a combination of line blot, ELISA and immunoprecipitation techniques, many of which were pioneered and developed during this period. From 2014 onwards, the EUROLINE Autoimmune Inflammatory Myopathies 16 antigen immunoblot (Euroimmun, Lübeck, Germany) was available in Salford and was used for autoantibody detection. These datasets were merged, a manual review of all patient records was undertaken and inclusion and exclusion criteria applied.

### Inclusion and exclusion criteria

The independent opinion on the certainty of an IIM diagnosis was obtained from at least two investigators and all definite IIM cases were eligible for inclusion. Clinical, serological and histological details were collected for these cases to allow for dense phenotyping and subsequent analysis. Cases were excluded if the expert consensus on diagnosis was uncertain, if the diagnosis was later found to be non-IIM, if the IIM had been initially diagnosed at an alternative institution, if substantial clinical information was missing or if the symptoms had started prior to the age of 18 years. Using all available information, including in some the later confirmation of myositis autoantibodies once these were available to clinicians in subsequent visits, these cases of expert opinion consensus definite incident IIM were classified into a single subgroup of PM, DM, ASS, IMNM, ADM, IBM or OM. This decision is informed by the authors’ understanding of current broad clinical consensus and more specifically influenced by the other relevant IIM classification criteria of Griggs (to define IBM cases), the European Neuromuscular Centre (especially for IMNM) and the suggested ASS criteria of Connors [13–15]. An overlap CTD was identified using the disease-specific consensus classification criteria, where available (i.e. for SSc, RA, SLE, SS), or on the basis of clinical opinion and serological status (in cases of MCTD) [16–21].

### Diagnosis of IIM by EULAR/ACR classification criteria

The EULAR/ACR criteria were applied to each individual case using the web calculator (available at www.imm.ki.se/biostatistics/calculators/iim). Cases were categorized using the suggested cut-points into possible IIM (score 5.3–5.4 without biopsy, 6.5–6.6 with biopsy; probability 50–54%), probable IIM (score 5.5–7.4 without biopsy, 6.7–8.6 with biopsy; probability 55–89%) and definite IIM (score ⩾7.5 without biopsy, ⩾8.7 with biopsy; probability ⩾90%). In the original paper, the ‘without biopsy’ cut-points were intended to be used only for subjects with a DM rash. However, for our study purposes we categorized all cases (DM and non-DM) both with and without biopsy data. The reported results include the biopsy data in subsequent analysis unless specifically stated.

In EULAR/ACR probable and definite IIM cases, the classification tree was used to assign an IIM subtype.

### Statistical methods

Statistical analyses were performed using SPSS version 23 (IBM, Armonk, NY, USA) and Excel 2010 (Microsoft, Redmond, WA, USA). Categorical variables are presented as numbers and percentages. Continuous variables with a normal distribution are presented as a mean with range and s.d. Sensitivity was calculated and presented with 95% CIs for both the probable and definite cut-points. Sensitivity, specificity and positive and negative predictive values for IIM subtype assignment are presented as a percentage with 95% CI. The agreement between expert opinion and classification criteria was assessed using Cohen’s κ coefficient, presented with the s.e.

## Results

### Case ascertainment

**Fig. 1:**
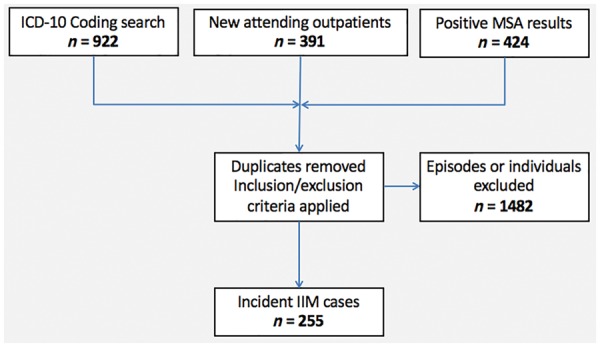
The three-stage case ascertainment process. ICD-10: International Classification of Diseases 10th Revision.

A total of 1637 potential cases were identified. After removal of duplicates, manual electronic patient record review and application of the inclusion and exclusion criteria, a cohort of 255 cases of expert-determined definite incident IIM was established (see Fig. 1 for details). All included cases had a minimum core set of data including a formal assessment of power (and distribution of weakness), a documented creatine kinase (CK) level, serological status, the presence or absence of dysphagia and biopsy status available to allow for the criteria to be applied. The more common underlying diagnoses in the excluded cases were inherited myopathies, such as a dystrophy or metabolic myopathy; primary neurological conditions with elevated CK levels, such as motor neurone disease; acquired causes of myositis that are not primarily immune mediated, such as toxic rhabdomyolysis or critical illness polyneuromyopathy; or simply an error in clinical episode coding. In addition, a substantial proportion of the MSA-positive cases were ultimately interpreted as false positives and excluded.

### Baseline characteristics

Overall, 157/255 (61.6%) cases were female and the mean age at IIM diagnosis was 55.3 years (range 21–84; s.d. 15.1). The proportion of the IIM subtypes assigned by expert opinion is summarized in Table 1, accompanied by the proportion of each subtype having biopsy data available. A total of 160/255 cases (62.7%) had full biopsy reports available, a further 19/255 (7.5%) had a detailed description of the biopsy findings in correspondence and 76/255 (29.8%) had no biopsy performed or available. In particular subtypes, such as IBM and IMNM, almost all cases had had biopsies performed with data available, whereas in others, such as ADM and ASS, biopsy frequency was substantially less. All cases had antibody results available and 112/255 (43.9%) cases were seronegative by routine SRFT serotyping. The antibody status of the remaining cases is detailed in Table 2. A total of 117/143 cases with an antibody had a single antibody detected. In the 26 remaining cases, the additional antibody was anti-Ro52 in 21 cases, anti-PmScl in 2 cases and anti-PL7, anti-SRP and anti-RNP in 1 case each. No case had more than two antibodies detected.

**Table 1:** Summary of expert-assigned IIM subtype and proportion of each with biopsy data available

Expert- assigned IIM subtype	Frequency, *n*	Total proportion of cohort, %	Biopsy available, *n* (%)
PM	37	14.5	30 (81.1)
DM	57	23.4	39 (68.4)
IBM	56	22.0	54 (96.4)
ADM	14	5.5	0 (0)
ASS	34	13.3	19 (55.9)
IMNM	25	9.8	25 (100)
OM	32	12.5	12 (37.5)

**Table 2:** Serum autoantibody frequency

	Antibody	Frequency, *n* (%) of total IIM cohort
ASS-specific	-Jo-1	26 (10.2)
	-PL-7	6 (2.4)
	-PL-12	1 (0.4)
	-EJ	1 (0.4)
DM-specific	-TIF1g	17 (6.7)
	-Mi-2	11 (4.3)
	-SAE	6 (2.4)
	-NXP2	5 (2.0)
	-MDA5	2 (0.8)
IMNM-specific	-HMGCoAR	10 (3.9)
	-SRP	7 (2.7)
Miscellaneous	-Ro52	38 (14.9)
	-PmScl	19 (7.5)
	-U1-RNP	10 (3.9)
	-Ku	6 (2.4)
	-Scl70	3 (1.2)
	-RNA polymerase III	1 (0.4)

### IIM diagnosis using EULAR/ACR criteria

The performance of the criteria, with expert consensus–defined IIM as the reference standard, using both the probable and definite cut-points (using biopsy data in all cases where available) is illustrated in Table 3.

**Table 3:** The diagnostic performance of the EULAR/ACR IIM classification criteria using the two criteria cut-points

EULAR/ ACR criteria cut-point	True positive	False negative	Sensitivity, % (95% CI)
Probable IIM (≥55% probability)	254	1	99.6 (97.2, 100)
Definite IIM (≥90% probability)	206	49	80.9 (76.0, 85.8)

#### The EULAR/ACR false-negative cases

The 49 cases that did not reach definite IIM probability with the classification criteria (Table 3) comprised 16 cases of IMNM, 12 OM, 9 PM, 7 IBM, 2 ASS, 2 DM and 1 ADM.

- IMNM. These comprised six HMGCo-AR-positive, three SRP-positive and seven seronegative cases, all of whom had a range of typical IMNM features on biopsy [14]. Four of 16 cases did not meet definite probability regardless of whether biopsy data were included. However, 12/16 did reach definite probability when the biopsy results were not included.
- OM. These comprised eight cases with concomitant SSc and four with MCTD. Only 4 of these 12 cases had biopsy data available, but again, when the biopsy findings were not included, 2/4 cases reached definite probability. In both cases the biopsies showed clear myopathic features but not the specific features relevant to the classification criteria.
- PM. All nine cases were seronegative and a biopsy was available for interpretation in seven. Two of the seven cases did not meet definite criteria regardless of whether biopsy information was included. However, the remaining five cases did meet definite criteria when the biopsy data were not included.
- IBM. Six of the seven cases had biopsy data available and five cases became definite when the biopsy findings were not included. These cases all had several features consistent with IBM on biopsy [multiple cyclooxygenase (COX)-negative fibres, p62-positive inclusions and widespread MHC-1 upregulation] alongside their IBM-suggestive clinical features (and met Griggs criteria for IBM), but the biopsies did not identify rimmed vacuoles, the specific feature relevant to the EULAR/ACR criteria [13].
- Others. Of the two ASS cases, one had no weakness [but did have interstitial lung disease (ILD), mechanics’ hands and polyarthritis with anti-Jo-1 and anti-Ro-52 autoantibodies] and a second had mild myositis but no biopsy performed. Of the two cases of DM missed by the EULAR/ACR criteria, one had an anti-Mi2 antibody, panniculitis and polyarthritis but no demonstrable myositis and no specific DM rash. The other DM case missed was seronegative but had skin ulceration, severe ILD and MRI evidence of myoedema in the absence of demonstrable weakness. The single case of ADM missed when employing this probability cut-point was a patient with Gottron’s papules and abnormal nailfold capillaroscopy, a positive anti-PmScl antibody but normal CK and no muscle biopsy performed.

The single case missed by the less stringent cut-point of probable was a case of seronegative PM with non-specific myopathic muscle biopsy findings. It became probable when the biopsy findings were not included.

#### IIM subtype assignment

The relationship between expert-assigned and criteria-assigned IIM subtype is shown in Table 4. The corresponding performance is summarized in Table 5.

**Table 4:** The relationship between expert-derived and classification criteria–derived IIM subtypes

		Classification tree–assigned IIM subtype				
		PM	DM	IBM	ADM	Totals
Expert-assigned IIM subtype	PM	37	0	0	0	37
	DM	3	54	0	0	57
	IBM	0	0	56	0	56
	ADM	0	0	0	14	14
	ASS	29	5	0	0	34
	IMNM	25	0	0	0	25
	OM	30	2	0	0	32
	Total	124	61	56	14	255

**Table 5:** Classification criteria performance when expert-assigned subtype is restricted to four categories specifically differentiated by the criteria

Classification criteria–assigned IIM subtype	Sensitivity, % (95% CI)	Specificity, % (95% CI)	Positive predictive value, % (95% CI)	Negative predictive value, % (95% CI)
PM	100 (100)	60.1 (53.6, 66.6)	29.8 (22.8, 37.9)	100 (100)
DM	94.7 (88.9, 100.5)	96.5 (93.9, 99.0)	88.5 (80.5, 96.5)	98.5 (96.7, 100.2)
IBM	100 (100)	100 (100)	100 (100)	100 (100)
ADM	100 (100)	100 (100)	100 (100)	100 (100)

The majority of cases [87/124 (70.2%)] subtyped as PM by the classification criteria were given an alternative diagnosis by expert opinion. Of the three cases that were classified as DM rather than PM by expert opinion, one had an anti-NXP2 antibody and calcinosis but no more specific DM cutaneous features and had no biopsy performed, one was seronegative but had perifascicular atrophy on biopsy and no rash and the final case was seronegative, had severe skin ulceration, severe ILD and MRI evidence of myoedema in the absence of demonstrable weakness and no biopsy was performed. Therefore all three cases represent the clinical entity of ‘DM *sine* dermatitis’. All cases of IBM and ADM were subtyped correctly by the classification tree. However, because the classification criteria do not include the ASS subtype, all these were classified differently between the two methods. The majority of ASS cases (29/34) were classified as PM, with the remaining five subtyped as DM because of their cutaneous manifestations. All expert-defined cases of IMNM were subtyped as PM by the EULAR/ACR criteria, as were the large majority of OM cases (30/32), except 2 patients classified as DM, both with anti-PmScl antibodies and EULAR/ACR defined SSc, myositis and Gottron’s papules or a heliotrope rash, respectively. Taken collectively, the κ correlation between expert-assigned and criteria-assigned subtype was 0.229 (s.e. 0.028). However, if the options for expert subtype assignment were restricted to PM, DM, IBM and ADM (excluding those not specifically included in the classification criteria), there was disagreement in only 3/255 cases [κ = 0.982 (s.e. 0.010)].

## Discussion

The EULAR/ACR classification criteria showed almost perfect sensitivity in identifying IIM in our cohort, with only one case not being identified as at least probable. This is despite the fact that in this study the criteria were applied at diagnosis, where one may postulate that the criteria will perform less well than in established disease given the reduced time for the full clinical manifestations to become apparent. These findings represent additional validation for the recently published EULAR/ACR criteria in an external patient cohort. The results also help extend their utility into a recently diagnosed cohort in addition to the more established IIM disease cohort used in the original validation process. However, when applying the more stringent probability cut-points for definite IIM, as proposed for most research purposes, the sensitivity of the EULAR/ACR criteria fell to 80.9%. The group of 49 cases missed using this cut-point had a range of clinical and serological phenotypes but in particular contained a disproportionate number of cases of IMNM and OM. Even though not currently validated for this purpose in non-DM patients, if available biopsy data were not included in the calculations of probability, a further 24 of the 34 (70.6%) cases reached definite probability scores, which would improve the overall sensitivity from 80.9 to 90.2% (range 86.5–93.8). This intriguing finding suggests that biopsy results reduce the utility of the criteria in these subtypes. Our study findings contrast somewhat with those of another publication responding to the primary publication, where they reported a sensitivity of 78.9% for the criteria in their cohort of 95 IIM cases [22]. The cause of these differences is unclear, but it highlights the importance of external validation in establishing the utility of newly developed criteria such as the EULAR/ACR criteria.

It is somewhat counterintuitive for classification criteria to perform with poorer sensitivity when more information is available, specifically in this case biopsy data, and we suggest several factors are at play here. First, muscle biopsy reports can be complex and challenging to reduce down to a binary decision between the presence or absence of a certain feature. Second, where the biopsy data reduced the probability of an IIM, the biopsies often showed myopathic features without other more specific findings. There is the potential for sampling error in biopsies, when either areas of affected muscle are inadvertently missed or the sample is obtained at a time when certain features may not be present (e.g. earlier in the course of IBM or in a patient already treated with immunosuppression). In clinical practice, non-specific biopsy findings do not make a diagnosis of IIM less likely in themselves. However, they do make a diagnosis of IIM less likely by default within the classification criteria because of consequently increased aggregate score cut-points when adding biopsy information. Finally, there are potential issues with the chosen biopsy criteria. In particular, the features associated with IMNM (e.g. pauci-immune appearances with occasional necrotic fibres), the perimysial-predominant pathology described in ASS or the IBM-suggestive features in addition to rimmed vacuoles (p62-positive inclusions, multiple COX-negative fibres, etc.) are not available to include in probability or subtype calculations [23]. Consequently it may be appropriate to consider not including biopsy data in patients with suspected IMNM or in those cases of ASS and IBM where the biopsy findings are non-specific during EULAR/ACR criteria calculations of IIM probability, as this appears to reduce their sensitivity.

Despite the excellent sensitivity of the criteria in diagnosing IIM, they did not correlate as well with expert opinion in IIM subtype assignment. As disagreement may arise even between expert clinicians, some differences would be anticipated. Within this context, however, the total of 94/255 (36.9%) cases that were subtyped differently by expert opinion *vs* classification criteria remains rather high and the potential clinical significance of such differences could be substantial; inaccurate subgroup assignment could have a large confounding effect on research outcomes. The two reasons identified for differing subtype assignment were either the capacity for expert opinion to incorporate additional clinically relevant information over that captured in the classification criteria and/or the use of additional subtypes not currently discriminated by the criteria. This conclusion is supported by the near-perfect correlation between expert opinion and criteria-assigned subtype when experts were restricted to only use the same four subtypes specifically included in the classification criteria. Expert opinion can incorporate the full range of available MSAs, the information from EMG and MRI, additional relevant biochemistry tests, a wider range of DM-associated cutaneous manifestations, nailfold capillaroscopy findings and the full range of biopsy features in arriving at a subtype conclusion. In addition, expert opinion can include other suggestive organ involvement, most notably ILD, which is not captured in the classification criteria. It may thus be appropriate for future research studies to include expert opinion IIM subtype in addition to classification criteria subtype to aid in the interpretation of findings.

A criticism of all previous IIM classification criteria has been the risk of overdiagnosis of PM, which is a small proportion of IIM diagnosed in clinical practice [2, 24, 25]. Our results here show the classification criteria diagnosed PM in 124/255 (48.6%) cases, whereas expert opinion diagnosed this in substantially less [37/255 (14.5%)]. Consequently, the specificity of the EULAR/ACR classification criteria for PM was only 60.1% with a positive predictive value of 29.3%. This criteria-assigned PM group contained patients with OM, ASS, IMNM and DM in decreasing order of frequency, in addition to cases agreed to represent PM. These data suggest that the EULAR/ACR classification criteria have not yet resolved the problem of PM overdiagnosis. Consequently, given the heterogeneity between subtypes in organ-specific manifestations, natural history and treatment responses, this issue may continue to muddy the waters of future research relying on these classification criteria for case definition.

Our cohort contains a relatively high proportion of patients with IBM. There are a number of factors that may have contributed to this, including some that are centre specific. Our centre sees a high proportion of tertiary referrals, which is a population that could feasibly be ‘enriched’ for IBM given its treatment refractory nature, and has also participated in a number of clinical trials in IBM over the study period, which may have confounded referral patterns. As discussed previously, the sensitivity of the criteria for early IBM in particular may be reduced compared with other subtypes and this may have impacted the overall performance. The criteria performed perfectly in assigning the IBM subtype, however, and this specificity is a clear strength for research purposes.

The limitations of this study include the use of a single-centre cohort, albeit one drawn from a large referral network, rather than a more diverse case population. The experts involved at our centre share similar opinions on the interpretation of case phenotypes, but this may not be representative of IIM experts more generally. However, the research strategy was chosen because it allowed for more complete data acquisition, negating a limitation of the classification criteria cohorts. The methodology for MSA detection changed during the study period once a commercially available autoantibody technique became available locally and the impact of this change on our serological results is uncertain. Expert opinion on IIM subtype incorporated all available clinical and histopathological information in addition to autoantibody profile and thus the impact of this uncertainty on our results would be anticipated to be minor. A strength of our study was application of the criteria at the time of diagnosis, earlier in the disease course than those used in the original validation cohorts and thus examining the utility of the criteria in a population of patients of importance to research. We utilized a multimodal case acquisition strategy, gathering consecutive cases over a 10 year period and therefore representing a real-world cohort rather than a preselected cohort of research study–enrolled IIM patients.

The EULAR/ACR IIM classification criteria performed almost perfectly in diagnosing IIM in our cohort, although with a reduced sensitivity when the more stringent definite cut-points were applied. There was disagreement between expert-assigned and criteria-assigned IIM subtype in 36.9% cases, in particular with a larger proportion of cases assigned as PM in the classification group (48.6%) *vs* the expert group (14.5%). The criteria-assigned PM group remains particularly heterogeneous and is important to consider in the conduct and interpretation of future IIM research studies.

## Supplementary Material

Supplementary Data
