# Supplementary material for: The performance of the European League Against Rheumatism/American College of Rheumatology idiopathic inflammatory myopathies classification criteria in an expert-defined 10 year incident cohort
Source: Rheumatology (Oxford). 2018 Nov 28;58(3):468–75. doi: 10.1093/rheumatology/key343 (PMC6381759; doi:10.1093/rheumatology/key343)
Supplement: Supplementary Data [file key343_supplementary_data.docx]

## Supplemental Data:

Table 6: ICD-10 code search terms used to screen inpatient episodes to identify incident idiopathic inflammatory myopathies.

| ICD-10 Code | Code descriptor |
| --- | --- |
| M33.1 | Other dermatomyositis |
| M33.2 | Polymyositis |
| M33.9 | Dermatopolymyositis, unspecified |
| M60.1 | Interstitial myositis |
| M60.8 | Other myositis |
| M60.9 | Myositis, unspecified |
| G72.4 | Inflammatory myopathy, not elsewhere classified |
| G72.8 | Other specified myopathies |
| G72.9 | Myopathy unspecified |
| G73.7 | Myopathy in other diseases specified elsewhere |
